# Combining Genotype Improvement and Statistical Media Optimization for Isoprenoid Production in *E. coli*

**DOI:** 10.1371/journal.pone.0075164

**Published:** 2013-10-04

**Authors:** Congqiang Zhang, Xixian Chen, Ruiyang Zou, Kang Zhou, Gregory Stephanopoulos, Heng-Phon Too

**Affiliations:** 1 Chemical and Pharmaceutical Engineering, Singapore-MIT Alliance, Singapore, Singapore; 2 Department of Biochemistry, National University of Singapore, Singapore, Singapore; 3 Department of Chemical Engineering, Massachusetts Institute of Technology, Cambridge, Massachusetts, United States of America; University of Groningen, The Netherlands

## Abstract

Isoprenoids are a large and diverse class of compounds that includes many high value natural products and are thus in great demand. To meet the increasing demand for isoprenoid compounds, metabolic engineering of microbes has been used to produce isoprenoids in an economical and sustainable manner. To achieve high isoprenoid yields using this technology, the availability of metabolic precursors feeding the deoxyxylulose phosphate (DXP) pathway, responsible for isoprenoid biosynthesis, has to be optimized. In this study, phosphoenolpyruvate, a vital DXP pathway precursor, was enriched by deleting the genes encoding the carbohydrate phosphotransferase system (PTS) in *E. coli*. Production of lycopene (a C40 isoprenoid) was maximized by optimizing growth medium and culture conditions. In optimized conditions, the lycopene yield from PTS mutant was seven fold higher than that obtained from the wild type strain. This resulted in the highest reported specific yield of lycopene produced from the DXP pathway in *E. coli* to date (20,000 µg/g dry cell weight). Both the copy number of the plasmid encoding the lycopene biosynthetic genes and the expression were found to be increased in the optimized media. Deletion of PTS together with a similar optimization strategy was also successful in enhancing the production of amorpha-1,4-diene, a distinct C15 isoprenoid, suggesting that the approaches developed herein can be generally applied to optimize production of other isoprenoids.

## Introduction

Isoprenoids constitute one of the most diverse classes of secondary metabolites in nature, with more than 55,000 distinct compounds [1]. The demand for some isoprenoids (e.g. artemisinin and taxol) is on the rise and there is a global shortage due to limited production from natural sources like plants. Market fluctuation and environmental changes have further exacerbated the problem and this has resulted in a growing demand for a sustainable supply of these isoprenoids [2], [3]. Metabolic engineering of microbes is an emerging solution that addresses these challenges [4].

**Figure 1:**
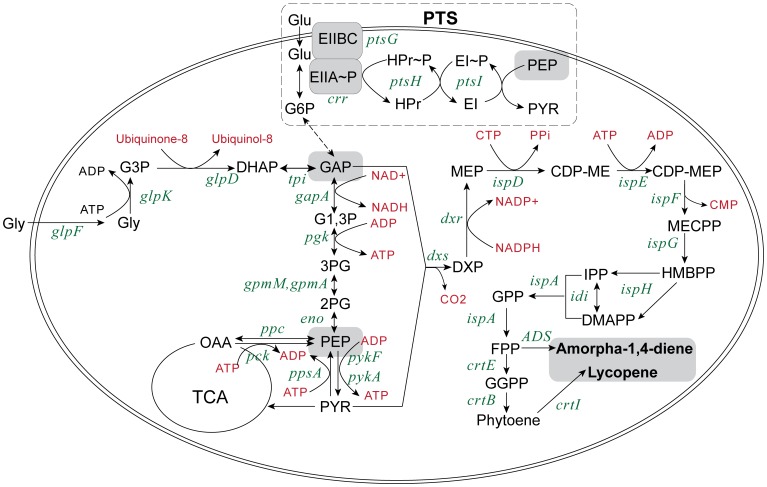
Relationship of the central metabolic and DXP pathways. The abbreviations for metabolites in the figure were as follows: glycerol (Gly), glucose (Glu), glucose-6-phosphate (G6P), glycerol-3-phosphate (G3P), dihydroxyacetone phosphate (DHAP), glyceraldehyde 3-phosphate (GAP), 1,3-biphospho-glycerate (G1,3P), 3-phospho-glycerate (3PG), 2-phospho-glycerate (2PG), phosphoenolpyruvate (PEP), the phosphotransferase system (PTS), pyruvate (PYR), oxaloacetate (OAA), tricarboxylic acid cycle (TCA), 1-deoxy-D-xylulose 5-phosphate (DXP), 2C-methyl-D-erythritol 4-phosphate (MEP), 4-diphosphocytidyl-2C-methyl D-erythritol (CDP-ME), 4-diphosphocytidyl-2C-methyl D-erythritol 2-phosphate (CDP-MEP), 2C-methyl-D-erythritol 2,4-diphosphate (MEC), hydroxylmethylbutenyl diphosphate (HMBPP), isopentenyl diphosphate (IPP) and dimethylallyl diphosphate (DMAPP), farnesyl pyrophosphate (FPP), geranylgeranyl pyrophosphate (GGPP), phosphate (PPi), carbohydrate phosphotransferase system (PTS). The abbreviations for enzyme-coding genes in the figure are as follows: PTS enzyme IIBC (*ptsG*), histidine protein (*ptsH*), PTS enzyme I (*ptsI*), PTS enzyme IIA(*crr*), glycerol facilitator (*glpF*), glycerol kinase (*glpK*), glycerol-3-phosphate dehydrogenase (*glpK*), triose phosphate isomerase (*tpi*), glyceraldehyde-3-phosphate dehydrogenase A (*gapA*), phosphoglycerate kinase (*pgk*), phosphoglycero mutase III (*gpmM*), phosphoglyceromutase I (*gpmA*), enolase (*eno*), PEP carboxylase (*ppc*), PEP carboxykinase (*pck*), phosphoenolpyruvate synthetase (*ppsA*), pyruvate kinase type I and II (*pykFA*), DXP synthase (*dxs*), DXP reductase (*dxr*), CDPME synthase (*ispD*), CDPME kinase (*ispE*), CDPMEP synthase (*ispF*), HMBPP synthase (*ispG*), HMBPP reductase (*ispH*), IPP isomerase (*idi*), farnesyl pyrophosphate (*FPP*) synthase (*ispA*), GGPP synthase (*crtE*), phytoene synthase (*crtB*) and phytoene desaturase (*crtI*) and amorpha-1,4-diene synthase (*ADS*).

In nature, all isoprenoids are derived from the common building blocks, isopentenyl diphosphate (IPP) and dimethylallyl diphosphate (DMAPP), which are synthesized by the 1-deoxy-D-xylulose 5-phosphate (DXP) pathway and/or the mevalonate pathway [1]. *E. coli* is one of the most frequently used microbes for isoprenoid production, using the native DXP pathway. In *E. coli*, phosphoenolpyruvate (PEP) is a vital precursor for the DXP pathway (Figure 1). An approach where central metabolism was genetically rerouted to increase PEP concentration was shown to be effective in enhancing the production of lycopene, an important isoprenoid compound [5]. As PEP is known to be consumed by the phosphotransferase system (PTS) when carbohydrates are imported [6], it is worth investigating whether the deletion of PTS would increase intracellular PEP concentration which may then result in the enhancement of isoprenoid production. However, deletion of PTS caused severe growth retardation when *E. coli* was grown in media containing glucose as the sole carbon source. This resulted in low cell density and a lower total yield of lycopene from the PTS knockout *E. coli*.

Optimization of the growth medium is an effective method to increase cell density and production of recombinant proteins [7], [8] and bulk chemicals [9], [10]. However, growth medium optimization has not been systematically investigated in metabolic engineering of microbes for the production of isoprenoids. A variety of standard media are commonly used for isoprenoid production without major modifications, including both complex media (LB [11], [12], 2xYT [13], [14], TB [15], [16]) and defined media (M9 [11], [17], 2xM9 [18], [19]). In medium optimization, it is usually impractical to test the full combinations of all the ingredients at different concentrations due to the relatively large number of possibilities. Statistically designing experiments to screen critical components, and optimizing the concentration of the screened components using response surface methodology (RSM) offers a unique opportunity to determine the optimal conditions with a minimal number of experiments.

In this study, the enhancement of lycopene production in *E. coli* was investigated by increasing the intracellular concentration of PEP using a PTS knockout mutant. PTS deletion resulted in low cell density in media containing only glucose as the carbon source. In order to increase cell density, the growth medium was then systematically optimized with the aid of factorial experiment design. Consequently, in addition to cell growth, the productivity of lycopene was also enhanced by medium optimization. The mechanism was subsequently investigated by metabolite and transcriptional profiling. In addition to the significant enhancement of lycopene production, amorpha-1,4-diene production was similarly enhanced by the use of the approach described herein, suggesting that the approach has a broader utility for the production of other isoprenoids.

## Results

### Increasing Lycopene Production and Biomass of PTS Mutant Strain

**Figure 2:**
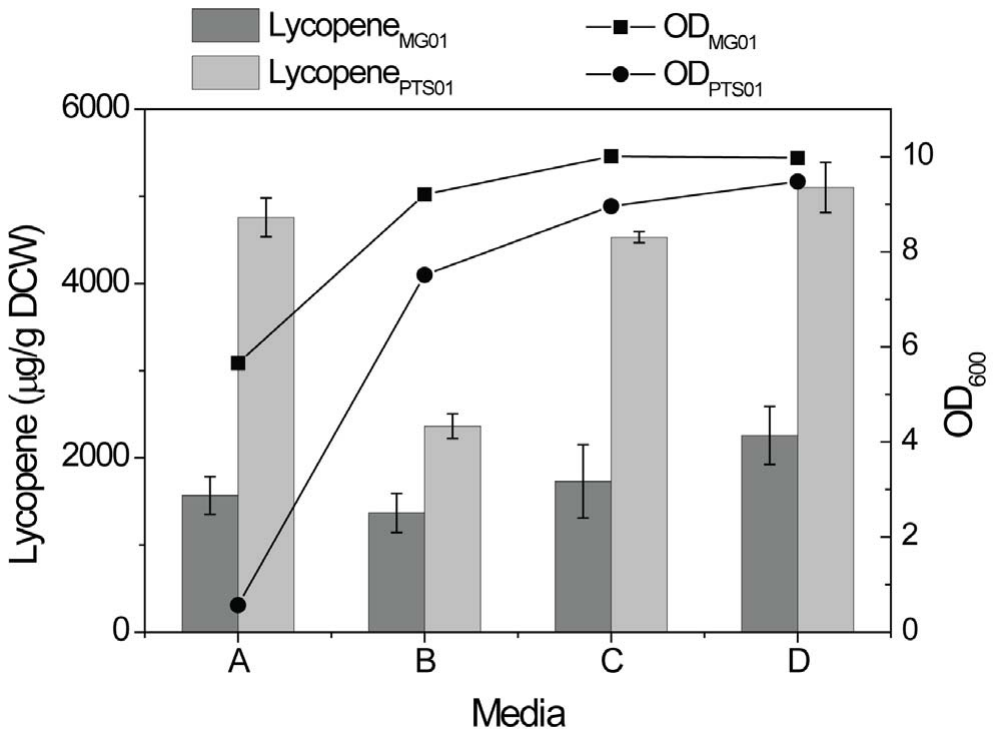
Comparison of lycopene yield and cell density of MG01 and PTS01 strains grown in different media compositions. Lycopene production and OD were assayed after 24[37] containing different concentrations of glucose and glycerol. The carbon concentrations in media A, B, C, D were 10 g/L glucose +0 g/L glycerol, 7 g/L glucose +3 g/L glycerol, 3 g/L glucose +7 g/L glycerol and 0 g/L glucose +10 g/L glycerol, respectively. All other components in the Riesenberg media were the same. The strains grew in the media at 37°C and 225 rpm).

PEP is a glycolytic intermediate and a major phospho-donor for many cellular processes. PEP can be converted to phosphoglycerates and then to glyceraldehyde 3-phosphate (GAP), the limiting precursor of the DXP pathway (Figure 1) [20]. It is reasonable that isoprenoid production can be enhanced through increasing the supply of PEP by deleting selective competing pathways, such as the phosphotransferase system (PTS). To test this hypothesis, the *ptsHIcrr* operon (encoding the components of the PTS) in *E. coli* genome was deleted, and the production of lycopene, a C40 isoprenoid, was compared in both the wild-type strain (MG01) and the knockout strain (PTS01). In line with the hypothesis, lycopene yield (g/g DCW) was increased by about three fold in PTS01 as compared to MG01 (Figure 2). However, the cell density was exceptionally low in the glucose minimal medium, consistent with previous reports [21]. This was likely due to the disruption of glucose uptake by PTS [22].

In order to improve the cell growth of PTS01, glycerol was added into the media to replace glucose. Cell density was found to increase with the addition of glycerol into the media, and the best growth was obtained when glycerol was used as the sole carbon source. More importantly, the specific productivity of lycopene (g lycopene/g DCW) in glycerol media was comparable to that of the media that had glucose as the sole carbon source. In addition, PTS01 produced more lycopene than MG01 in the glycerol media (Figure 2).

### Statistical Optimization of Growth Media for the PTS Mutant Strain

A systematic optimization of the growth medium for PTS01 was performed to increase lycopene productivity. The contributions of five ingredients to lycopene productivity were characterized using a minimal run resolution IV fractional factorial design (Table 1 and Table S1 in File S1), based on the glycerol minimal medium optimized for high cell density [23]. The five factors chosen for optimization are: Glycerol, KH_2_PO_4,_ (NH_4_)_2_HPO_4,_ sodium pyruvate, and L-arabinose. Glycerol was the major carbon source in media; KH_2_PO_4_ served as a buffer, ionic agent and phosphorus source; (NH_4_)_2_HPO_4_ was the source of both nitrogen and phosphorus; sodium pyruvate was used as the substrate of the DXP pathway for lycopene production; and L-arabinose was used to induce the pBAD vector for the expression of *dxs*-*idi*-*ispDF* operon.

**Table 1:** Summary of selected medium ingredients for screening (Details of experiment design are shown in Table S1 in File S1).

Factor	Name	Unit	Low (−)	High (+)	Function in media
A	Glycerol	g/L	8	20	C source
B	KH_2_PO_4_	g/L	8	20	P source, buffer
C	(NH_4_)_2_HPO_4_	g/L	2	5	N source, buffer
D	Na(Pyruvate)	g/L	2	5	Precursor
E	L-Arabinose	mM	0	0.1	Inducer

**Figure 3:**
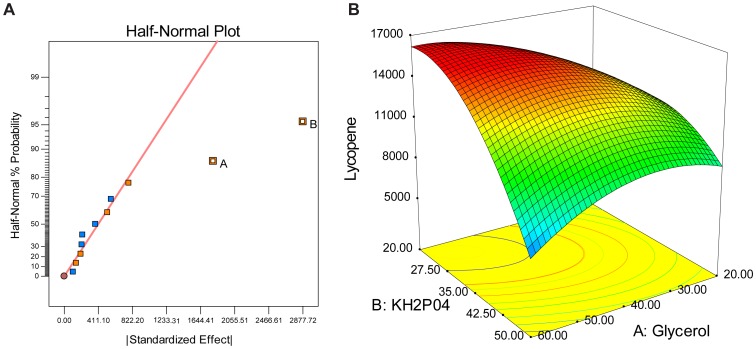
Identification and optimization of critical media composition for the production of lycopene in PTS01 strain. (A) Half-normal probability plot of the results of minimal resolution IV experiment design (The details of the experiment design were shown in Table S1 in File S1). In the half-normal probability plot, the line was called the near zero line. The estimated effect of an unimportant factor will typically be on or close to a near-zero line, while the estimated effect of an important factor will typically be displaced well off the line. (B) RSM plot of lycopene production versus concentrations of glycerol and KH_2_PO_4_ of PTS01.

The results of experimental design showed that glycerol and KH_2_PO_4_ were the most important factors for lycopene production (Figure 3A) and the relationships can be described by Equation 1 (Factor A is glycerol and factor B is KH_2_PO_4_). The equation indicated that lycopene production was enhanced by increased concentrations of glycerol and KH_2_PO_4_.
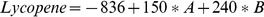
(1)


After identifying the two critical ingredients in media for lycopene production, the optimal concentrations were further determined by applying RSM with a central composite design (Table S2 in File S1). The experimental results were analyzed statistically using ANOVA. The mean predicted and observed responses, and the details of the statistical analysis, are presented in Table S2 in File S1 and Table S3 in File S1. The values of the regression coefficients were calculated and the fitted equation (in terms of coded values) for the prediction of lycopene production (µg/g DCW) are shown in Equation 2, where Factor A is glycerol and factor B is KH_2_PO_4_.
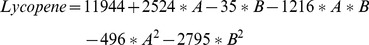
(2)


Based on this equation, optimal concentrations of glycerol and KH_2_PO_4_ for production of lycopene (16,261 µg/g DCW) were predicted to be 50 g/L and 24 g/L, respectively (Figure 3B). To verify the model, lycopene production was measured from *E. coli* grown in these predicted optimal concentrations of glycerol and KH_2_PO_4._ The measured lycopene yield was 17542±1105 µg/g DCW (Table 2), similar to the predicted optimized yield of 16,261 µg/g DCW, thereby validating the model.

**Table 2:** Model prediction and experimental validation of RSM.

Item	Glycerol (g/L)	KH_2_PO_4_ (g/L)	Lycopene (ppm)
Model Prediction	50	24	16261
Verifying Experiment	50	25	17542±1105
R-Squared	0.95		
Adj R-Squared	0.90		

**Figure 4:**
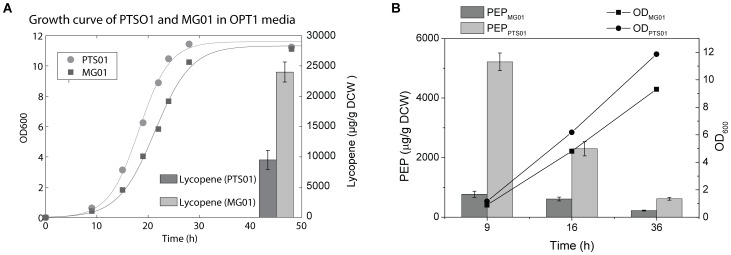
Comparison of lycopene production, growth curve and PEP levels of MG01 and PTS01. (A) Growth curve and lycopene productions of PTS01 and MG01 in optimal glycerol medium (OPT1) at 37°C, with a shaking speed of 300 rpm. (B) Comparison of PEP levels in MG01 and PTS01 at different growth stages (early-log phase, pre-induction, 9 h; mid-log phase, 16 h post-induction; stationary phase, 36 h post-induction). Both of the strains were grown in OPT1 at 37°C, with a shaking speed of 300 rpm).

As shown in Figure 4A, PTS01 grown in this optimized media (designated OPT1) produced more than twice as much lycopene as MG01. PEP concentrations were consistently higher in PTS01 than MG01 at all growth phase (Figure 4B). There is a correlation between lycopene production and PEP concentration, suggesting that increased lycopene production can be attributed to higher amounts of PEP available.

**Figure 5:**
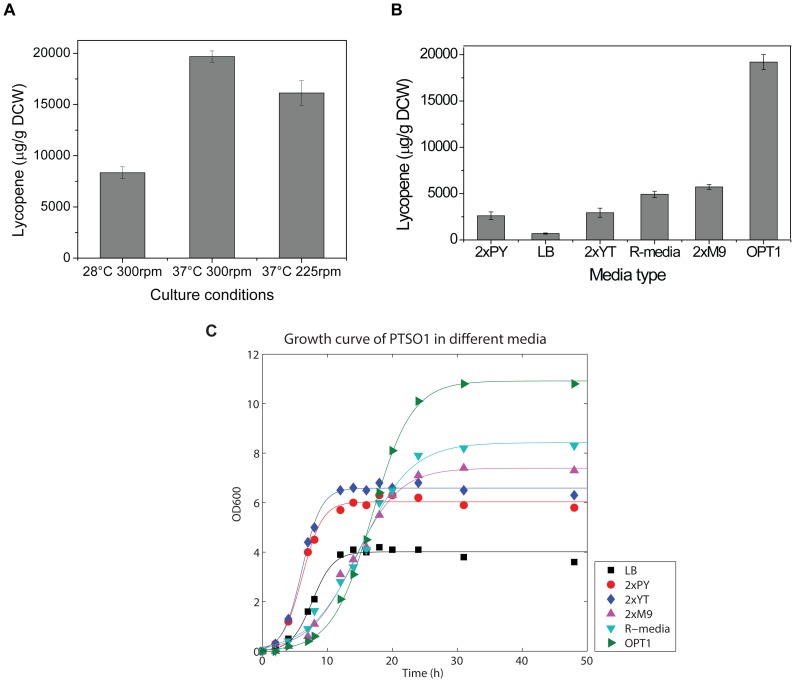
Optimization of culture conditions for PTS01 and comparison of OPT1 with other commonly used media. (A) Lycopene production of PTS01 at different temperatures and shaking speeds. (B) Comparison of lycopene production of PTS01 in 2xPY, LB, 2xYT, 2xM9, R-media and OPT1 media. The strain was grown at 37°C, with a shaking speed of 300 rpm. (C) The growth curves of PTS01 in 2xPY, LB, 2xYT, 2xM9, R-media and OPT1 media.

The culture conditions of temperature and O_2_ feeding (controlled by rotation speed of incubation) were optimized. It was found that by increasing rotation speed to 300 rpm from 225 rpm, and keeping culture temperature at 37°C (earlier experiments were conducted at 225 rpm and 37°C), lycopene yield was increased to around 20,000 µg/g DCW for PTS01 (Figure 5A).

The optimized medium (OPT1) and culture conditions were used in further experiments to investigate the effect of PTS deletion on the accumulation of intracellular PEP and the concomitant increase in lycopene productivity.

The growth rate and lycopene productivity of cells grown in OPT1 were compared with other commonly used complex medium (2xPY, LB, 2xYT) and defined medium (2xM9 with 10 g/L glycerol, R-media with 10 g/L glycerol). It was found that the optimized medium, OPT1, was superior in lycopene production (Figure 5B) compared to all other media tested. Although the strain grew faster in complex media (LB, 2xYT and 2xPY), the biomass yields were lower when compared to the defined media, including OPT1 (Figure 5C). By using this approach to optimize lycopene production using the DXP pathway, we achieved the highest lycopene yield reported so far, compared to previous studies [19], [24].

### Transcriptional Investigation and Metabolites Profiling of PST01 Grown in OPT1 Medium

Transcriptional analysis by qPCR has been used to gain mechanistic insights into metabolic engineering [25], [26]. The transcription of the genes involved in lycopene biosynthesis was investigated in order to better understand the underlying mechanisms affecting lycopene production in the OPT1 medium, and how it compares to complex media. The PTS01 strain harbored two plasmids (pBAD-SIDF and pAC-LYC) which overexpressed four rate-limiting genes of the DXP pathway (*dxs*, *idi* and *ispDF*) and three heterologous lycopene synthetic genes (*crtE*, *crtB* and *crtI*), respectively (Figure 1).

PTS01 grown in complex media (2xPY) and the OPT1 medium was measured and compared using the expression level of one gene from each plasmid (*dxs* and *crtE*) and an endogenous DXP pathway gene (*ispE*). Interestingly, at different growth phases, the transcriptional levels of the plasmid-encoded genes (*dxs* and *crtE*) were found to be up-regulated by approximately 4–8 times in the OPT1 medium, compared to those in 2xPY (Figure S1 in File S2). Similarly, the plasmid copy numbers of both of these plasmids were up-regulated in the OPT1 medium (Figure S2 in File S2). However, the expression level of the endogenous gene (*ispE*) was similar in both strains when grown in either media (Figure S1 in File S2). These results indicate that increased transcriptions of *dxs* and *crtE* were probably due to the increase in plasmid copy number. Therefore, it was hypothesized that the OPT1 medium increased the plasmid copy numbers of pBAD-SIDF and pAC-LYC in greater quantities than the 2xPY medium, and this led to the increased expression levels of the genes in those plasmids, and finally contributed to the increased production of lycopene.

**Figure 6:**
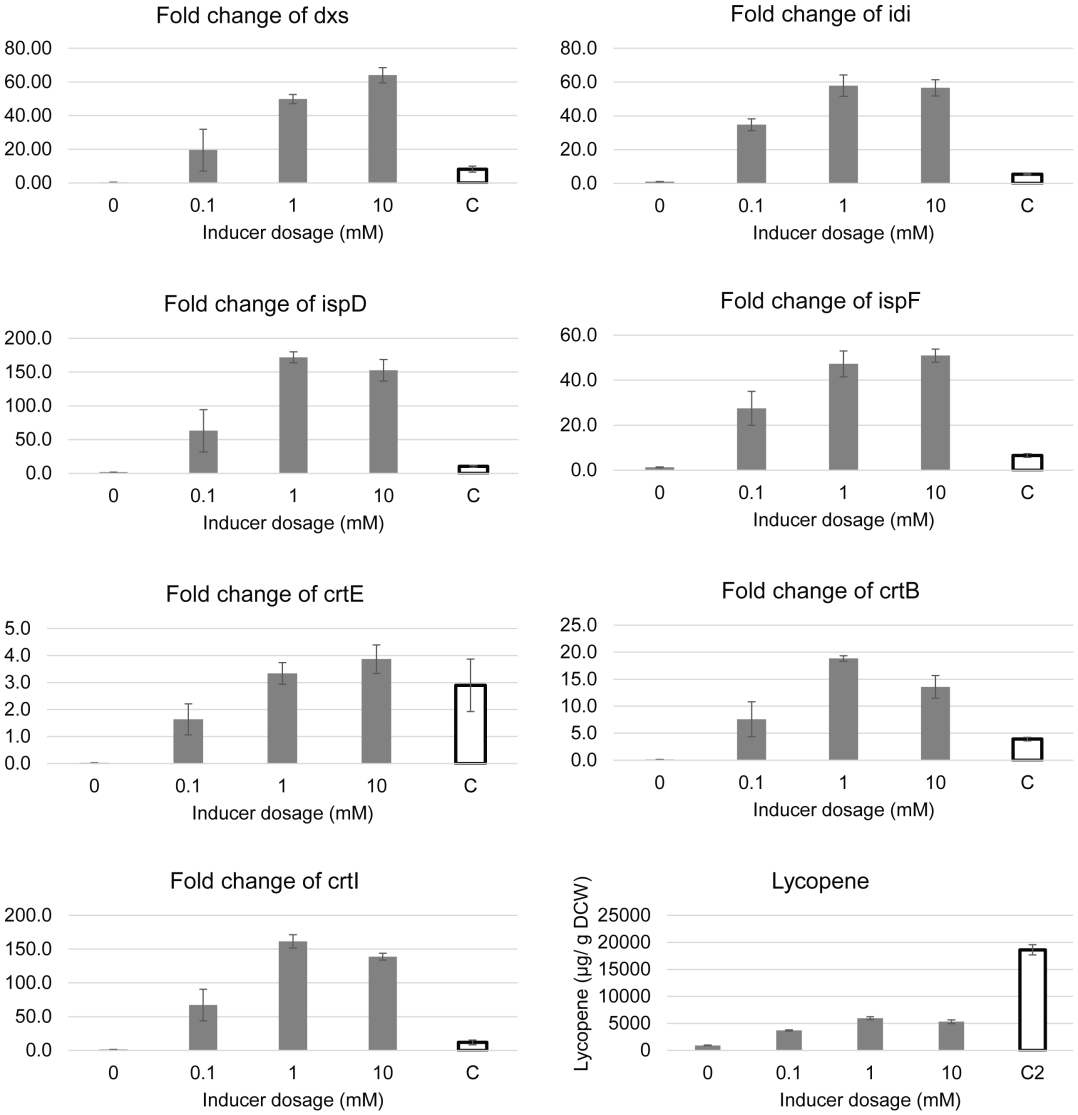
Transcriptional analysis of *dxs*, *idi*, *ispD ispF*, *crtE*, *crtB* and *crtI*. PTS02 was grown in 2xPY and induced with different concentrations of L-arabinose (0.1, 1 and 10 mM). Columns labelled “C” represent the expression levels of PTS01 grown in OPT1 in the absence of the inducer (L-arabinose). Fold changes of transcriptional levels of these genes were normalized to the expression levels of corresponding genes of PTS01 grown in 2xPY in the absence of the inducer (L-arabinose).

To test the hypothesis that co-upregulations of these genes were major contributors to the enhancement of lycopene production in the OPT1 medium, both the operon *dxs*-*idi*-*ispDF* and the operon *crtEBI* were overexpressed in 2xPY. Using the pBAD vector, the operon *dxs*-*idi*-*ispDF* was put under the control of the araBAD promoter. The lycopene biosynthetic genes (*crtEBI)* were placed under the control of the araBAD promoter in the pAC vector (designated pACM-LYC, which was constructed from plasmid pAC-LYC by replacing the constitutive promoter with the araBAD inducible promoter). PTS02, a MG1655 PTS^-^ strain carrying pACM-LYC and pBAD-SIDF was then generated and grown in 2xPY media. By gradually increasing the concentration of L-arabinose, the transcriptional levels of *dxs*-*idi*-*ispDF* and *crtEBI* were induced to levels greater than or similar to that found in PTS01 in the OPT1 medium (Figure 6). Lycopene production was found to increase gradually from 500 to 6000 µg/g DCW with increasing amounts of L-arabinose, indicative of the enhancement of lycopene production by the co-upregulation of *dxs*-*idi*-*ispDF* and *crtEBI.* However, despite achieving transcriptional levels of *dxs*-*idi*-*ispDF* and *crtEBI* of PTS02 (grown in 2xPY) that were higher or at least comparable with those of PTS01 (grown in OPT1 medium), the maximal lycopene production of PTS02 in 2xPY (∼6000 µg/g DCW) was nonetheless found to be significantly lower than that of PTS01 in OPT1 (∼18,000 µg/g DCW) (Figure 6). These results indicated that the co-upregulation of *dxs-idi-ispDF* and *crtEBI* was essential but not sufficient to enhance lycopene production to the levels observed in PTS01 grown in the OPT1 medium.

In addition to transcriptional analysis, metabolite profiling also provides useful information in attempts to understand underlying biochemical mechanisms [27]. The intracellular metabolites of the PTS01 cells grown in the 2xPY and OPT1 media were profiled and compared. A panel of metabolites were found to be significantly enriched in the cells grown in the OPT1 medium, which included glycerol-3-phosphate (G3P), DXP and pyrophosphate (Figure S3 in File S2). G3P and DXP are critical intermediates in the isoprenoid synthetic pathway when routing from glycerol (the carbon source of the defined medium), and the increases in exponential state concentrations were consistent with enhanced lycopene production. The upregulated level of pyrophosphate was also expected in the OPT1 medium, as the medium contained large amounts of phosphate. Major cellular co-factors ATP and NADH were detected in different growth stages and most of their concentrations were comparable in the 2xPY and OPT1 media, suggesting that the increase in lycopene production was not due to changes in these cofactor supplies in the OPT1 medium.

### Production of Amorpha-1,4-diene in PTS03 Strain in Defined Media

**Figure 7:**
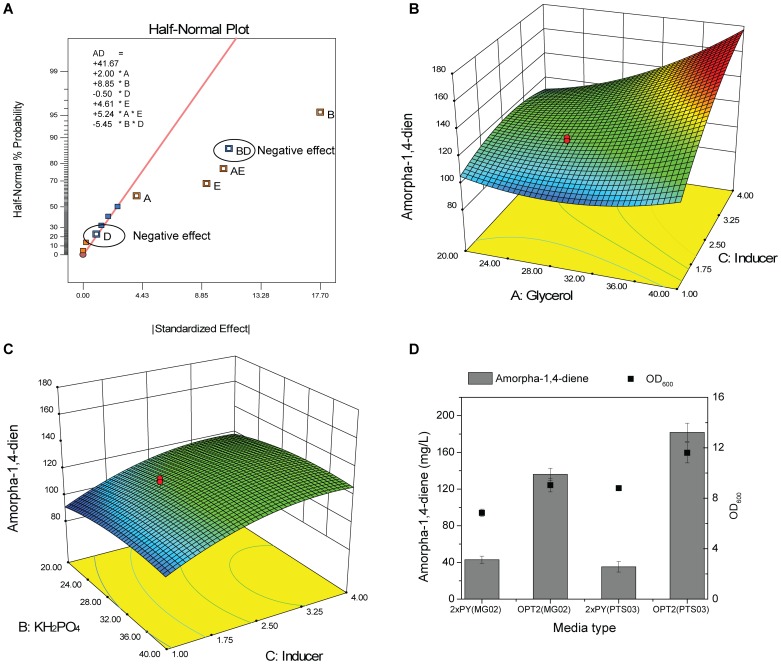
Media optimization of amorpha-1,4-diene production in PTS03 strain. (A) Screening of key factors for amorpha-1,4-diene production (The factors share the same denotations as those described in Figure 3A. In the figure, A, B and E are dominant factors with positive effects on the production of amorpha-1.4-diene, therefore they were selected as key factors for further optimization). (B) Production of amorpha-1,4-diene versus glycerol concentration and inducer dosage. (C) Production of amorpha-1,4-diene versus KH_2_PO_4_ concentration and inducer dosage. (D) Comparison of amorpha-1,4-diene production in 2xPY and OPT2 media for the MG02 and PTS03 strains. The details of the experiment design are described in Supplementary Table S6 and S7 in File S1.

Using PTS deletion and media development, we have established a rapid and effective method to increase lycopene production in *E. coli*. In order to demonstrate the broader utility of this approach, the production of amorpha-1,4-diene, a distinct C15 isoprenoid, was attempted. The growth medium was optimized using the same statistical method as described before, where three important factors A, B and E (which are glycerol, KH_2_PO_4_ and L-arabinose, respectively) were screened by fractional factorial design (Figure 7A and Table S4 in File S1). The optimal concentrations of the three critical factors were determined by RSM, and the results are shown in Figure 7B, Figure 7C and Table S5 in File S1. This optimized medium for amorpha-1,4-diene was designated OPT2 and an enhancement of amorpha-1,4-diene production was observed (182 mg/L), which was significantly higher than the yield derived from 2xPY complex media (35.3 mg/L). Similarly to lycopene, the production of amorpha-1,4-diene (182 mg/L) was better in the PTS^-^ strain (PTS03) than in the wide type (MG02) in OPT2 medium (136 mg/L, Figure 7D).

## Discussion

In this study, the deletion of PTS and the use of statistical medium optimization enhanced the production of a C40 isoprenoid (lycopene) and a C15 isoprenoid (amorpha-1,4-diene), suggesting that the approaches developed herein may be used for optimizing the production of other isoprenoids.

Increasing intracellular concentration of PEP in PTS knockout mutant when grown in glucose media has been reported to enhance the yield of aromatics [21], [22]. However, it is not known if PTS deletion can also enrich intracellular PEP when cells are grown in glycerol media. Glycerol uptake genes (*glpF*, *glpK* and *glpD*) are known to be regulated by carbon catabolite repression in *E. coli* and are intimately linked to Enzyme IIA (gene *ccr,* part of the *ptsHIcrr* operon) and the ratio of PEP to pyruvate [28]–[30]. Although it is expected that PTS deletion will affect transcription of PTS related genes (e.g. *pck*, *ppc*, *ppsA*, *pykFA* and *eno,* Figure 1), no significant difference was found in the expression levels of these PEP related genes in PTS01 and MG01 (Figure S4 in File S2). This does not rule out the possibility that PTS disruption may modulate post-translational events resulting in PEP accumulation, an interesting possibility yet to be examined. PEP is thought to transfer phosphate to EI (gene ptsI), a PEP-dependent protein-kinase. The phosphate group is subsequently transferred from EI∼P to HPr (gene ptsH), from HPr∼P to the soluble EIIAGlc (sometimes also called EIIACrr), and finally from EIIAGlc∼P to the glucose-specific membrane protein EIICBGlc (gene ptsG), resulting in the uptake and phosphorylation of glucose (Figure 1) [30]. As there are more than 20 different Enzymes II in *E. coli* [6], the deletion of the *ptsHIcrr* operon in this study may cause a decrease in phosphorylation of these enzymes resulting in the accumulation of PEP. This hypothesis is the subject of future investigations.

PTS knockout not only affects the uptake of glucose but also plays an important role in carbon catabolite repression. This is because phosphorylated EIIA^Glc^ binds and activates adenylate cyclase (AC), which leads to cyclic AMP (cAMP) synthesis, and high cAMP concentrations trigger the formation of cAMP–CRP (cyclic AMP (cAMP) receptor protein; also called catabolite gene-activator protein (CAP)) complexes, which bind and activate the promoters of many catabolic genes [30]. It was also reported that the araBAD promoter was affected by CAP [31]. To understand the effects of PTS knockout on the activities of the araBAD promoter in the pBAD-SIDF and a constitutive promoter in pAC-crtEBI, the expression levels of genes in the pBAD-SIDF plasmid and pAC-crtEBI plasmid for the MG01 strain and PTS01 strain, both cultivated in OPT1 media, were compared. However, the results indicated that the expression levels of all the genes in the two plasmids were not affected significantly when PTS was deleted (Figure S5 in File S2). This could be because there was no arabinose added into the system, so the activity of the araBAD promoter was relatively low under such conditions and the effects of a PTS knockout was insignificant.

The optimization of a growth medium is an effective method to increase cell density and production of recombinant proteins [7], [8] and bulk chemicals [9], [10]. To optimize the media, it was impractical to test the full combinations of components at different concentrations. Furthermore, it is a challenge to construct a mechanistic model to predict the interactions of the medium compositions with respect to the production of isoprenoids. Instead, we explored the use of statistically designed experiments [32] to screen critical components and optimize the concentrations rapidly, with a minimal number of experiments. From such studies, glycerol and KH_2_PO_4_ were identified as the most dominant factors in lycopene production, while pyruvate, (NH_4_)_2_HPO_4_ and inducer dosage were found to be less critical. It is likely that glycerol and KH_2_PO_4_ have global metabolic effects. Limiting the nitrogen supply increased the production of amorpha-1,4-diene [33], which is consistent with our results that (NH_4_)_2_HPO_4_ is not critical for the production of both lycopene and amorpha-1,4-diene. Pyruvate was determined to be less critical to the production of such isoprenoids, perhaps because of an abundance of intracellular pyruvate.

Interestingly, the effects of inducer dosage were different for the production of lycopene and amorpha-1,4-diene. For lycopene, low inducer dosage did not affect production and higher inducer dosage was detrimental to production in PTS01 (data not shown). While there was an optimal inducer dosage for amorpha-1,4-diene production, too much or too little inducer had a negative impact. In addition to media composition, temperature and oxygen supply are also important factors that were tuned to optimize the final yield of lycopene.

Our transcriptional analysis provided useful insights into how optimization of media resulted in increased lycopene production. The increase in *dxs* and *crtE* expressions was observed in OPT1 when compared to 2xPY (Figure S1 in File S2). Further investigation indicated that the upregulation of these genes was due to greater plasmid copy numbers in OPT1 (Figure S2 in File S2). It is known that media composition can affect the quality and yield of plasmids [34]. Similarly, the alteration of carbon-to-nitrogen (C:N) ratio [35] and amino acid starvation [36] have also been shown to increase plasmid copy numbers. By lowering the specific growth rate, plasmid DNA yields can be increased [37]. This inverse correlation between plasmid DNA synthesis and growth rate was thought to be due to higher plasmid stability as well as increased plasmid replication in reduced growth rates [38]. The PTS deleted strain (PTS01) grew slowly in the OPT1 medium, which is consistent with the earlier proposal, and it may share similar mechanisms to increase the plasmid copy number, thereby increasing transcription.

Although lycopene production levels increased significantly, paralleling an increase in the transcription of *dxs*-*idi*-*ispDF* and *crtEBI* in 2xPY, the highest lycopene yield from 2xPY was still much less than in OPT1. These results suggest that the co-upregulation of *dxs*-*idi*-*ispDF* and *crtEBI* was required to enhance lycopene production, but was still insufficient to reach the levels observed in PTS01 grown in an OPT1 medium. Metabolite profiling results showed that major cellular co-factors ATP and NADH were comparable in the 2xPY and OPT1 media, suggesting that the availability of cofactors was unlikely to be a limitation and may not account for the increase in lycopene production in the OPT1 medium. In addition to the transcriptional upregulation of *crtEBI*, the prolonged exponential phase of OPT1 could be beneficial in diverting more carbon fluxes into DXP pathway, thus increasing the overall yield.

## Conclusion

To our knowledge, this is the first study that demonstrated the enhancement of isoprenoid production (lycopene and amorpha-1,4-diene) using the phosphotransferase system deficient strain of *E. coli.* The growth medium was rapidly optimized for the knockout mutant with the aid of statistical experiment design. This concurrently overcome cell growth retardation, a side effect of PTS disruption on a glucose medium, and increased the production of lycopene, a C40 isoprenoid. The highest lycopene yield in this study was 20,000 µg/g DCW, which is a significant yield compared to published reports. An attempt to understand the underlying mechanisms in cells grown in the optimized defined media demonstrated that the transcription of the lycopene biosynthetic genes was essential but insufficient to account for the observed enhancements in lycopene production. Deletion of PTS in combination with statistical medium optimization was also successfully applied to enhance the production of amorpha-1,4-diene, a distinct C15 isoprenoid, suggesting that the approach developed herein has broad utility beyond the scope of this study.

## Materials and Methods

### Statistical Experimental Design

Fractional factorial designs of experiment and response surface methodology were calculated using Design Expert® V8 Software, Stat-Ease, Inc.

### Bacteria Strains and Plasmids

*E coli.* K-12 MG1655 [F- lambda- *ilvG*- *rfb*-50 *rph*-1] was used as the parental strain, and MG1655 PTS^-^ (PTS^-^ for short) was used in the following experiments for lycopene production with the pAC-LYC plasmid [39]. PTS^-^ strain was obtained by replacing operon *ptsHIcrr* with a kanamycin-resistant gene using the λ Red recombination system developed by Datsenko et al. [40]. The details of the oligos (EcoRB-PTSF-KanF and EcoRBR-PTSR-KanR) and the plasmid pKD46 used for this work was shown in Table S6 in File S1, and the correct mutant of PTS^-^ was verified by PCR with the wild-type strain as the control. Plasmid pBAD-SIDF was constructed via ligation of *dxs*-*idi*-*ispDF* operon amplified by polymerase chain reaction (PCR) from p20T7MEP [41] into a modified pBAD-B vector (Invitrogen), which controlled the expression of the *dxs*-*idi*-*ispDF* operon using the araBAD promoter. Plasmid pACM-LYC was constructed by modifying the pAC-LYC plasmid so that three genes *crtE*, *crtB* and *crtI* were under the control of the araBAD promoter. Plasmid pAC-ADS was constructed based on pAC-LYC by replacing the crtEBI operon with the amorpha-1,4-diene synthase gene (ADS) using the ligase-independent cloning (LIC) method [42]. All the information about the strains, plasmids and primers is in Table S6 in File S1.

### Culture Media and Growth Conditions

1. Defined media recipe for optimization

Different concentrations of glycerol, (NH_4_)_2_HPO_4_, KH_2_PO_4_, sodium pyruvate and citric acid were added into the media according to the experimental design. In addition, the media also contained 0.5 g/L MgSO_4_, 2.5 mg/L CoCl_2_·6H_2_O, 15.0 mg/L MnSO_4_·4H_2_O, 1.5 mg/L CuSO_4_·2H_2_O, 3 mg/L H_3_BO_3_, 2.5 mg/L Na_2_MoO_4_·2H_2_O, 13 mg/L Zn(CH_3_COO)_2_, 60 mg/L Fe(III) citrate, 4.5 mg/L thiamine·HCl and 8.4 mg/L EDTA.

The final optimized defined media for PTS01 consisted of 50 g/L Glycerol, 24 g/L KH_2_PO_4_, 4 g/L (NH_4_)_2_HPO_4_, 1.7 g/L citric acid, and all the other components were kept at the same levels as the Riesenberg media [23]. This optimized defined medium was named OPT1.

The final optimized defined media for PTS03 consisted of 45 g/L Glycerol, 27.5 g/L KH_2_PO_4_, 4 g/L (NH_4_)_2_HPO_4_, 1.7 g/L citric acid, and all the other components were the same as the Riesenberg media [23]. This optimized defined medium was named OPT2. The concentration of L-arabinose (inducer) in OPT2 was 5 mM.

All the defined media were adjusted to pH7.0 using a NaOH solution, and filtered with a 0.2 micron membrane.

2. Riesenberg media (R-media) composition [23]

The R-media contained 10 g/L glycerol, 4 g/L (NH_4_)_2_HPO_4_, 13.3 g/L KH_2_PO_4_,1.7 g/L citric acid, 2.5 mg/L CoCl_2_·6H_2_O,15.0 mg/L MnSO_4_·4H_2_O, 1.5 mg/L CuSO_4_·2H_2_O, 3 mg/L H_3_BO_3_, 2.5 mg/L Na_2_MoO_4_·2H_2_O, 13 mg/L Zn(CH_3_COO)_2_, 60 mg/L Fe(III) citrate, 4.5 mg/L thiamine·HCl and 8.4 mg/L EDTA.

Glycerol, (NH4)2HPO4, KH2PO4 and citric acid were autoclaved separately. Sterile solutions of glucose, MgSO4, and thiamine-HCl were added afterwards, the pH of the media was adjusted to 7.0, and the media was autoclaved at 121°C for 20 mins.

3. 2xM9 defined media composition [19]

The 2xM9 media contained 25.6 g/L Na2HPO4·7H2O, 6 g/L KH2PO4, 1 g/L NaCl, 2 g/L NH4Cl, 2 mM MgSO4, 0.1 mM CaCl2 and 10 g/L glycerol. The pH of the media was adjusted to 7.0, and the media was autoclaved at 121°C for 20 mins.

4. 2xPY, LB and 2xYT complex media compositions

The 2xPY media contained 20 g/L peptone, 10 g/L yeast extract and 10 g/L NaCl. The pH of the media was adjusted to 7.0 and the media was autoclaved at 121°C for 20 mins.

The LB media contained 10 g/L tryptone, 5 g/L yeast extract and 10 g/L NaCl. The pH of the media was adjusted to 7.0 and the media was autoclaved at 121°C for 20 mins.

The 2xYT media contained 16 g/L tryptone, 10 g/L yeast extract and 5 g/L NaCl. The pH of the media was adjusted to 7.0 and the media was autoclaved at 121°C for 20 mins.

1% (v/v) overnight grown cell culture was inoculated into 1 mL 2xPY or defined medium in a 14 mL BD Falcon™ tube. Cells were grown at 37°C (or 28°C) with 300 rpm (or 225 rpm) shaking, and induced with L-arabinose when OD600 reached the range of 0.5∼1.0. The media were supplemented with 100 mg/L Ampicillin and 34 mg/L chloramphenicol to maintain the plasmids pBAD-SIDF and pAC-LYC (or pBAD-LYC) respectively. Besides, 25 mg/L kanamycin was added into the PTS01, PTS02 and PTS03 strain. For the strain that was producing amorpha-1,4-diene, the cells were grown at 28°C in order to decrease the evaporation of dodecane.

Cell density was monitored spectrophotometrically at 600 nm.

### Lycopene and Amorpha-1,4-diene Assay

Intracellular lycopene content was extracted from 10–100 µL bacterial culture (depending on the content of lycopene in the cells) in the stationary phase (24 h for LB, 2xYT and 2xPY, 48 h for 2xM9 and other defined media after induction). Cell pellets were washed with PBS, and lycopene was extracted by acetone. The lycopene content was quantified through absorbance at 472 nm using a 96-well microplate reader (Spectra Max 190, Molecular Devices) and concentrations were calculated by interpreting the standard curve. Cell mass was calculated by correlating DCW with a reading of OD600 and used for calculating specific productivity (µg lycopene/g DCW).

The strains producing amopha-4,11-diene were cultured in defined media with the addition of another 25% (v/v) organic dodecane phase to extract amopha-4,11-diene [16]. Amopha-4,11-diene was extracted by diluting 5 µL dodecane phase into 495 µL ethyl acetate and analyzed on an Agilent 7980A gas chromatograph equipped with an Agilent 5975C mass spectrometer (GC/MS), by scanning at only 189 m/z ion (single-ion monitoring, SIM mode), using trans-caryophyllene as the internal standard. The temperature program for the GC/MS analysis was essentially as described [33].

### RNA Purification and cDNA Synthesis

Total RNA from *E. coli* was prepared using TRIzol® reagent (Invitrogen) according to the manufacturer’s instructions. Total RNA was collected from samples in quadruplicate at each treatment time point. RNA concentration was quantified using a NanoDrop ND-1000 spectrophotometer (Thermo Scientific). Eight hundred ng of total RNA were reverse transcribed in a total volume of 20 µL containing ImpromII (Promega) for 60 min at 42°C according to the manufacturer’s instructions. The reaction was terminated by heating at 70°C for 10 min.

### Quantitative Real-time PCR

The cDNA levels were analyzed using a BioRad iCycler 4 Real-Time PCR Detection System (Bio-Rad) with SYBR Green I detection. Each sample was measured in duplicate in a 96-well plate (Bio-Rad) in a reaction mixture (25 µL final volume) containing 1x XtensaMix-SG (BioWORKS, Singapore), 200 nM primer mix, 2.5 mM MgCl_2_, 0.75 U of iTaq DNA polymerase (iDNA). Real time PCR was performed with an initial denaturation of 3 min at 95°C, followed by 40 cycles of 20 s at 95°C, 20 s at 60°C, and 20 s at 72°C. The primers used for real time PCR were given in supplementary data, and the reference gene used for normalization of real time PCR data was *cysG* [43].

The plasmid copy number was obtained by normalizing the copy number of plasmid resistant genes (ampicillin resistant gene for pBAD-SIDF and chloramphenicol resistant gene for pAC-LYC) to the copy number of chromosomal gene *ispE*. Standard curves were constructed using linearized plasmids.

### Metabolites Assay by LC-MS

Metabolites were analyzed on the UPLC (Waters ACQUITY UPLC) – (TOF) MS (Bruker micrOTOF II) platform. Cell pellets were collected at a density of ∼1 OD by centrifugation at 16,000 *g* for 1 min. The supernatant was then removed and the cell pellet was re-suspended in 30 uL of double distilled water. 120 uL of 100% methanol was then added to the cell suspension and incubated at room temperature for 10 min. The solution was centrifuged at 16,000 *g* for 2 min, and 50 uL of the clear supernatant was collected for analysis. Five uL of the clarified supernatant was injected into the reverse phase UPLC C18 column (Waters CSH C18 1.7 µm, 2.1 mm×50 mm) and separation was carried out at a flow rate of 0.15 mL/min with an aqueous mobile phase consisting of 15 mM acetic acid and 10 mM tributylamine. Elution was carried out with methanol, in an increasing concentration gradient, as shown in Table S7 in File S1. Electrospray ionization was used and (TOF) mass spectrometry setting was essentially as described [44].

## Supporting Information

File S1 Table S1, Experimental design of Min Run Res IV for the production of lycopene of PTS01 strain. Table S2. Central composite design of RSM design for production of lycopene of PTS01 strain with corresponding results. Table S3, Analysis of RSM design of PTS01 strain. Table S4, Experimental design of Min Run Res IV for the production of amorpha-1,4-diene of PTS03 strain. Table S5, Central composite design of RSM design for the production of amorpha-1,4-diene of PTS03 strain. Table S6, *E. coli* strains, plasmids and oligonucleotides used in this study. Table S7, Mobile phase gradient used for the separation of DXP intermediates.
(DOC)

File S2 Figure S1, Fold change of transcriptional levels of dxs, ispE and crtE in PTS01 strain grown in OPT1 as compared to those in 2xPY medium and lycopene production. The transcriptional levels measured in 2xPY and OPT1 were compared at four different growth stages, late lag phase (0 h for 2xPY and 0 h for OPT1, the time of induction was set as time 0 h), early log phase (2 h for 2xPY and 4 h for OPT1), middle log phase (4 h for 2xPY and 10 h for OPT1) and late log phase (4 h for 2xPY and 10 h for OPT1). The sampling time in each growth stage was based on differences in the growth rates of PTS01, which was faster in 2xPY than in OPT1 (As shown in Figure 5.C). All the measurements were normalized to the expression of *cysG*. Figure S2, Fold change of plasmid copy number in PTS01 grown in OPT1 and 2xPY media. The x-axis described parameters identical to Figure S1 in File S2. The plasmid copy number was calculated using the copy number of a plasmid resistant gene (ampicillin resistant gene for pBAD-SIDF and chloramphenicol resistant gene for pAC-LYC) normalized by the copy number of the chromosomal gene *ispE*. Figure S3, Concentrations of metabolites and cofactors in PTS01 strain grown in 2xPY or OPT1 media. The metabolites in 2xPY and OPT1 were compared at three different growth stages, early log phase (3 h for 2xPY, 6 h for OPT1), middle log phase (6 h for 2xPY, 12 h for OPT1) and early stationary phase (12 h, 24 h). The time of induction was defined as time 0 h. Figure S4, Fold change of transcriptional levels of genes involved in the metabolic pathway of glycerol in PTS01 and MG01 grown in OPT1 media. The growth media used in this study was OPT1 and all the samples of MG01 and PTS01 were collected at the early log phase. The expression levels of all the genes were normalized to *cysG.* Figure S5, Fold change of transcriptional levels of genes in the pBAD-SIDF and pAC-crtEBI pathway of glycerol in PTS01 and MG01 grown in OPT1 media. The growth media used in this study was OPT1 and all the samples of MG01 and PTS01 were collected at the early log phase. The expression levels of all the genes were normalized to *cysG*.
(DOC)
