# Supplementary material for: Combining Genotype Improvement and Statistical Media Optimization for Isoprenoid Production in E. coli
Source: PLoS One. 2013 Oct 4;8(10):e75164. doi: 10.1371/journal.pone.0075164 (PMC3790805; doi:10.1371/journal.pone.0075164)
Supplement: File S1 — Table S1, Experimental design of Min Run Res IV for the production of lycopene of PTS01 strain. Table S2. Central composite design of RSM design for production of lycopene of PTS01 strain with corresponding results. Table S3, Analysis of RSM design of PTS01 strain. Table S4, Experimental design of Min Run Res IV for the production of amorpha-1,4-diene of PTS03 strain. Table S5, Central composite design of RSM design for the production of amorpha-1,4-diene of PTS03 strain. Table S6, E. coli strains, plasmids and oligonucleotides used in this study. Table S7, Mobile phase gradient used for the separation of DXP intermediates. (DOC) [file pone.0075164.s001.doc]

**File 1**

Table S1. Experimental design of Min Run Res IV for the production of lycopene of PTS01 strain

| **No.** | **Glycerol/ (g/L)** | **KH2PO4/ (g/L)** | **(NH4)2HPO4/ (g/L)** | **Pyruvate (g/L)** | **Inducer/ mM** | **Lycopene/ (µg/g DCW)** |
| --- | --- | --- | --- | --- | --- | --- |
| 1 | 8 | 8 | 5 | 5 | 0 | 1992 |
| 2 | 8 | 8 | 5 | 2 | 0 | 1755 |
| 3 | 20 | 8 | 2 | 2 | 0 | 2705 |
| 4 | 8 | 20 | 5 | 5 | 0.1 | 4688 |
| 5 | 20 | 8 | 5 | 5 | 0 | 4694 |
| 6 | 20 | 20 | 2 | 2 | 0.1 | 7304 |
| 7 | 20 | 8 | 5 | 2 | 0.1 | 4337 |
| 8 | 8 | 20 | 2 | 5 | 0 | 4851 |
| 9 | 20 | 20 | 5 | 2 | 0 | 6473 |
| 10 | 8 | 20 | 2 | 2 | 0.1 | 5444 |
| 11 | 8 | 8 | 2 | 5 | 0.1 | 3578 |
| 12 | 20 | 20 | 2 | 5 | 0.1 | 7568 |

Table S2. Central composite design of RSM design for production of lycopene of PTS01 strain with corresponding results

| **Order** | **Glycerol/ (g/L)** | **KH2PO4/ (g/L)** | **Observed lycopene/ (µg/g DCW)** | **Predicted lycopene/ (µg/g DCW)** |
| --- | --- | --- | --- | --- |
| 1 | 10 | 20 | 5602 | 4947 |
| 2 | 30 | 20 | 11365 | 12429 |
| 3 | 10 | 50 | 8184 | 7308 |
| 4 | 30 | 50 | 9083 | 9925 |
| 5 | 5.9 | 35 | 6258 | 7380 |
| 6 | 34.1 | 35 | 15831 | 14522 |
| 7 | 20 | 13.8 | 6654 | 6404 |
| 8 | 20 | 56.2 | 6240 | 6303 |
| 9 | 20 | 35 | 13331 | 12222 |
| 10 | 20 | 35 | 12142 | 12222 |
| 11 | 20 | 35 | 12107 | 12222 |
| 12 | 20 | 35 | 11733 | 12222 |
| 13 | 20 | 35 | 11797 | 12222 |

Table S3. Analysis of RSM design of PTS01 strain for the production of lycopene

| **Source** | **Coefficient Estimate** | **p-value** |
| --- | --- | --- |
| Model |  | 0.001 |
| A-Glycerol | 2525 | 0.0003 |
| B-KH2P04 | -36 | 0.9261 |
| A*B | -1216 | 0.0540 |
| A^2^ | -635 | 0.1551 |
| B^2^ | -2934 | 0.0002 |

Table S4. Experimental design of Min Run Res IV for the production of amorpha-1,4-diene of PTS03 strain

| **Glycerol/ (g/L)** | **KH2PO4/ (g/L)** | **(NH4)2HPO4/ (g/L)** | **Pyruvate (g/L)** | **Inducer/ mM** | **AD /(mg/L)** |
| --- | --- | --- | --- | --- | --- |
| 8 | 8 | 5 | 5 | 0.1 | 33.6 |
| 8 | 8 | 5 | 2 | 0.1 | 26.3 |
| 20 | 8 | 2 | 2 | 0.1 | 19.6 |
| 8 | 20 | 5 | 5 | 0.3 | 40.4 |
| 20 | 8 | 5 | 5 | 0.1 | 29.4 |
| 20 | 20 | 2 | 2 | 0.3 | 69.2 |
| 20 | 8 | 5 | 2 | 0.3 | 40.4 |
| 8 | 20 | 2 | 5 | 0.1 | 46.3 |
| 20 | 20 | 5 | 2 | 0.1 | 49.5 |
| 8 | 20 | 2 | 2 | 0.3 | 52.1 |
| 8 | 8 | 2 | 5 | 0.3 | 38.4 |
| 20 | 20 | 2 | 5 | 0.3 | 55.0 |

Table S5. Central composite design of RSM design for the production of amorpha-1,4-diene of PTS03 strain

| **Order** | **Glycerol/ (g/L)** | **KH2PO4 /(g/L)** | **Inducer/ mM** | **Observed AD/ (mg/L)** | **Predicted AD / (mg/L)** |
| --- | --- | --- | --- | --- | --- |
| 1 | 27.5 | 27.5 | 2 | 118.9 | 115.92 |
| 2 | 35 | 35 | 3 | 158.9 | 147.25 |
| 3 | 27.5 | 14.9 | 2 | 101.7 | 100.64 |
| 4 | 35 | 20 | 3 | 139.0 | 136.4 |
| 5 | 35 | 35 | 1 | 102.2 | 105.72 |
| 6 | 27.5 | 27.5 | 2 | 98.5 | 115.92 |
| 7 | 20 | 20 | 1 | 90.0 | 99.56 |
| 8 | 27.5 | 27.5 | 3.7 | 111.3 | 126.64 |
| 9 | 35 | 20 | 1 | 92.8 | 94.87 |
| 10 | 27.5 | 27.5 | 2 | 118.9 | 115.92 |
| 11 | 40.1 | 27.5 | 2 | 140.2 | 143.03 |
| 12 | 27.5 | 40.1 | 2 | 107.8 | 115.85 |
| 13 | 20 | 20 | 3 | 122.8 | 111.46 |
| 14 | 20 | 35 | 3 | 122.8 | 118.7 |
| 15 | 20 | 35 | 1 | 112.1 | 106.79 |
| 16 | 27.5 | 27.5 | 2 | 119.1 | 115.92 |
| 17 | 14.9 | 27.5 | 2 | 118.7 | 122.97 |
| 18 | 27.5 | 27.5 | 2 | 121.6 | 115.92 |
| 19 | 27.5 | 27.5 | 2 | 119.6 | 115.92 |
| 20 | 27.5 | 27.5 | 0.3 | 90.0 | 81.71 |

Table S6. E. coli strains, plasmids and oligonucleotides used in this study

|  | **Relevant characteristics** | **Reference** |
| --- | --- | --- |
| **E coli strains** |  |  |
| MG01 | K12 MG1655 carrying plasmid pBAD-SIDF and plasmid pAC-LYC | This work |
| MG02 | K12 MG1655 carrying plasmid pBAD-SIDF and plasmid pAC-ADS | This work |
| PTS01 | Originated from MG1655, with ∆p*tsHIcrr*::kan, carrying plasmid pBAD-SIDF and plasmid pAC-LYC | This work |
| PTS02 | Originated from MG1655, with ∆p*tsHIcrr*::kan, carrying plasmid pBAD-SIDF and plasmid pACM-LYC | This work |
| PTS03 | Originated from MG1655, with ∆p*tsHIcrr*::kan, carrying plasmid pBAD-SIDF and plasmid pAC-ADS | This work |
| **Plasmids** |  |  |
| pAC-LYC | crtE, crtI, crtB genes from *E*.*herbicola* in pACYC184,Cm^R^ |  |
| pACM-LYC | Inducible araBAD promoter, crtE, crtI, crtB genes from *E*.*herbicola* in pACYC184,Cm^R^ | This work |
| pKD-46 | Red recombinase expression plasmids, Am^R^ |  |
| pBAD-SIDF | Containing *dxs*, *idi*, *ispD* and *ispF* genes, Cm^R^ | This work |
|  |  |  |
| **Primers** |  |  |
| EcoRB-PTSF-KanF | GTGGCCTGCTTCAAACTTTCGCCCCTCCTGGCATTGATTCAGCCTGTCGGTAGCTTGCAGTGGGCTTACA | |
| EcoRBR-PTSR-KanR | GCGGATAACCGGGGTTTCACCCACGGTTACGCTACCGGACAGTTTGATCAGGATGCCGACGGATTTG | |
| ILIC-ADS(PAC)f | GG*GTA*AC*GG*AAT*TGT*GAGCGGATAAC (* stands for phosphorothioate oligonucleotide) | |
| ILIC-PAC(ADS)r | AC*AAT*TC*CGT*TAC*CC*GCTGAATTGTCT | |
| ILIC-ADS(PAC)r | TCA*GG*TT*TTA*GA*GGA*TGGACATTGGGT | |
| ILIC-PAC(ADS)f | CC*TC*TA*AA*ACC*TGA*TCCGCCAGAAT | |
|  |  | |
| dxs-F | CGGCTATCACTATAACGATGG | |
| dxs-R | CACGACGCTTCACAATGC | |
| ispE-F | GGAAGCGGTGCGAATATC | |
| ispE-R | ATTGCCAGAGATGATTTAATGC | |
| crtE-F | GTAAAGCGGGCGTTTCG | |
| crtE-R | GCCAGCAGCATCAGC | |
| idi-F | TGTATTACACGGTATTGATGCCACG | |
| idi-R | AGCTGGGTAAATGCAGATAATCGTT | |
| ispD-F | GCAACCACTCATTTGGATGTTTGC | |
| ispD-R | CACCGAGTGTTCAAGAATGGTTTG | |
| ispF-F | CGAATTGGACACGGTTTTGACG | |
| ispF-R | CATCGGTCAACGCATGGAGC | |
| crtB-F | GTGATGGGCGTGCGGGATGA | |
| crtB-R | AGCCACTCGGCGGGCAGATA | |
| crtI-F | GACCAGGGCTTTACCTTTGACG | |
| crtI-R | CAGCAGAGTCGGTAGAAGGGTTTT | |
| cysG-F | TTGTCGGCGGTGGTGATGTC | |
| cysG-R | ATGCGGTGAACTGTGGAATAAACG | |
| amp-F | TTTTTGCTCACCCAGAAACGCTG | |
| amp-R | CGGGGCGAAAACTCTCAAGGATCTT | |
| cam-F | CTGGAGTGAATACCACGACG | |
| cam-R | GGATTGGCTGAGACGAAAA | |
| tpiA-F | GATGTGGCTGCCTTCA | |
| tpiA-R | TTCACGAGCTGGTTTCT | |
| gapA-F | TATCGGTCGCATTGTT | |
| gapA-R | CGTCCCATTTCAGGTT | |
| pgk-F | ATACCGTGAGTAGAAGCC | |
| pgk-R | AAGACGACGAAACCCT | |
| gpmM-F | GATACCGCCGTTGCTG | |
| gpmM-R | ACGCCACCGTTGAAGA | |
| gpmA-F | GCTCGTCGCCATACTTT | |
| gpmA-R | CGCTATCCATACCCTGTG | |
| eno-F | GTTACGCCTTTACCCA | |
| eno-R | ACTGTTGAAGCCGAAG | |
| ppsA-F | CACGGCAAGCAGGTTA | |
| ppsA-R | TTTCGCCCACTCAATAT | |
| pykF-F | GCACTGACCACCAACG | |
| pykF-R | GAGCCAGTTCTTTACCC | |
| pykA-F | ATTACTAACCCGATGCC | |
| pykA-R | GTTTCTGACGGATACTGC | |
| ppc-F | CATCACTCCCGCATCTT | |
| ppc-R | CGCTGTTTGAAACCCTC | |
| pck-F | ACAAAGGCAAAGGTAAGA | |
| pck-R | GGACGGAAAGACGAGTA | |

*The primers were designed based on genomic sequence of *Escherichia coli str. K-12 substr. MG1655*, the genbank accession number is NC_000913.2

# Literature

1. Cunningham FX, Jr., Sun Z, Chamovitz D, Hirschberg J, Gantt E (1994) Molecular structure and enzymatic function of lycopene cyclase from the cyanobacterium Synechococcus sp strain PCC7942. Plant Cell 6: 1107-1121.

2. Datsenko KA (2000) One-step inactivation of chromosomal genes in Escherichia coli K-12 using PCR products. Proceedings of the National Academy of Sciences 97: 6640-6645.

Table S7. Mobile phase gradient used for the separation of DXP intermediates.

| **Step** | **Accumulative Time (mins)** | **Aqueous solution*** | **Methanol** |
| --- | --- | --- | --- |
| 1 | 1.8 | 100% | 0 |
| 2 | 3.1 | 60% | 40% |
| 4 | 4.9 | 60% | 40% |
| 5 | 5.4 | 10% | 90% |
| 6 | 9.5 | 10% | 90% |
| 7 | 10 | 100% | 0 |

*Aqueous solution: 15mM acetic acid and 10mM tributylamine
