# Supplementary material for: Combining Genotype Improvement and Statistical Media Optimization for Isoprenoid Production in E. coli
Source: PLoS One. 2013 Oct 4;8(10):e75164. doi: 10.1371/journal.pone.0075164 (PMC3790805; doi:10.1371/journal.pone.0075164)

**File 2**

Figure S1. Fold change of transcriptional levels of dxs, ispE and crtE in PTS01 strain grown in OPT1 as compared to those in 2xPY medium and lycopene production.

The transcriptional levels measured in 2xPY and OPT1 were compared at four different growth stages, late lag phase (0h for 2xPY and 0h for OPT1, the time of induction was set as time 0h), early log phase (2h for 2xPY and 4h for OPT1), middle log phase (4h for 2xPY and 10h for OPT1) and late log phase (4h for 2xPY and 10h for OPT1). The sampling time in each growth stage was due to differences in growth rates of PTS01 which was faster in 2xPY than in OPT1 (As shown in **Figure 5.C**). All the measurements were normalized to the expression of *cysG*.


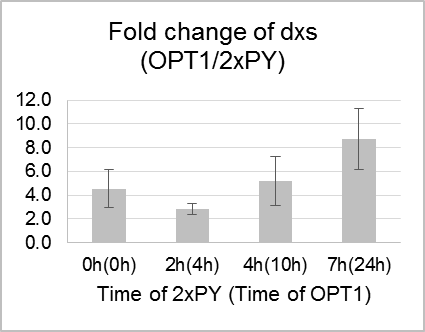

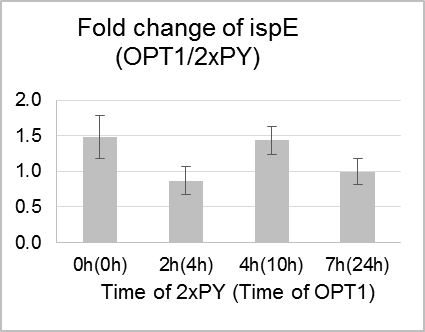

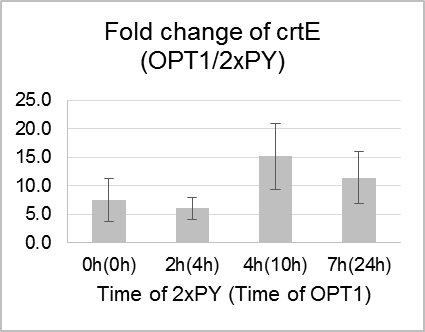

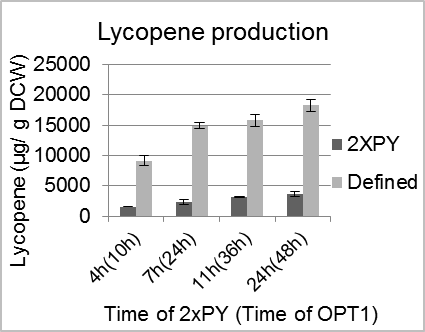


Figure S2. Fold change of plasmid copy number in PTS01 grown in OPT1 and 2xPY media

The x-axis described parameters identical to Figure S1. Plasmid copy number was calculated using copy number of plasmid resistant gene (ampicillin resistant gene for pBAD-SIDF and chloramphenicol resistant gene for pAC-LYC) normalized by the copy number of chromosomal gene *ispE*.


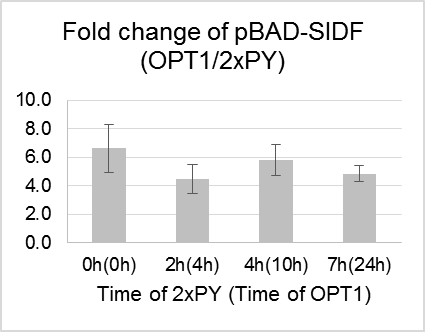

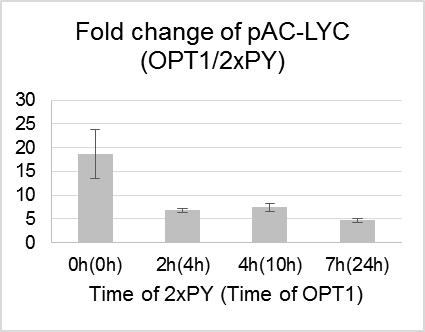


Figure S3. Concentrations of metabolites and cofactors in PTS01 strain grown in 2xPY or OPT1 media.

The metabolites in 2xPY and OPT1 were compared at three different growth stages, early log phase (3h for 2xPY, 6h for OPT1) , middle log phase (6h for 2xPY, 12h for OPT1) and early stationary phase (12h, 24h). The time of induction was defined as time 0h.


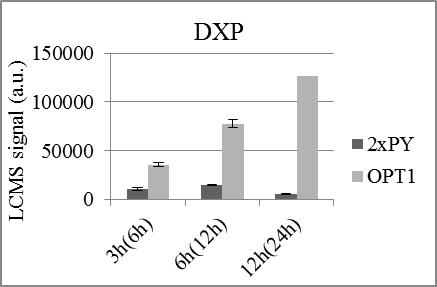

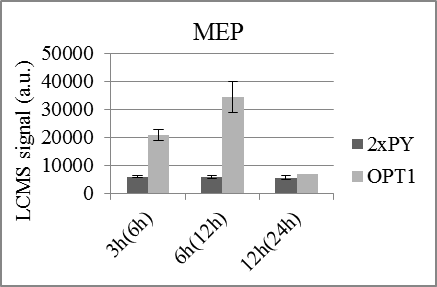

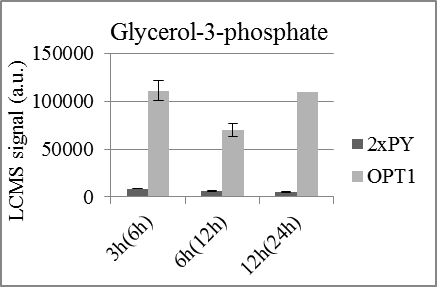

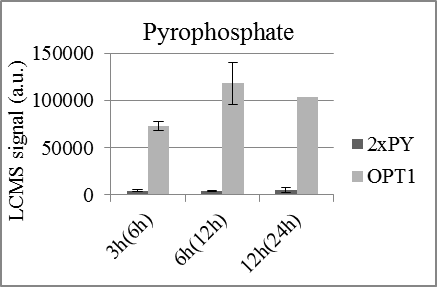

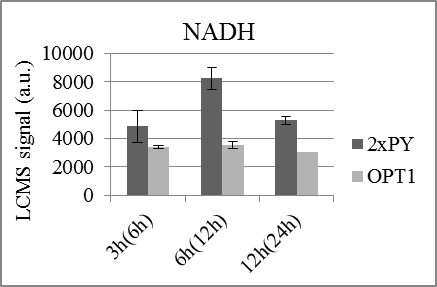

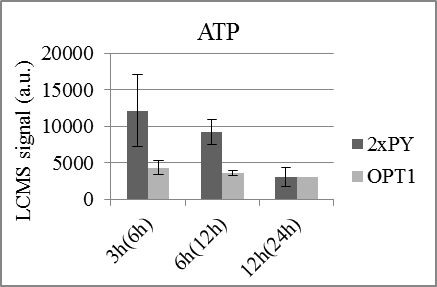

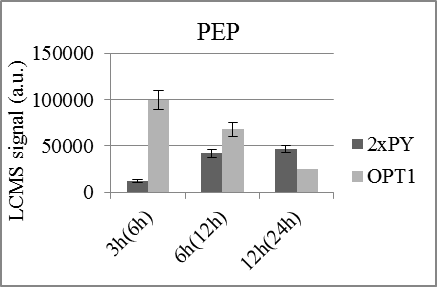

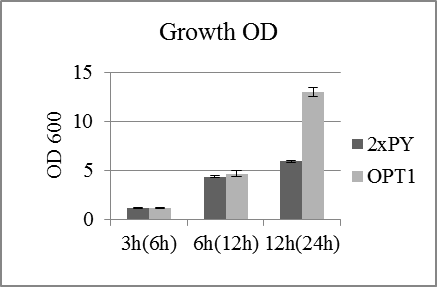


Figure S4. Fold change of transcriptional levels of genes involved in the metabolic pathway of glycerol in PTS01 and MG01 grown in OPT1 media.

The growth media used in this study was OPT1 and all the samples of MG01 and PTS01 were collected at early log phase. The expression levels of all the genes were normalized to *cysG*


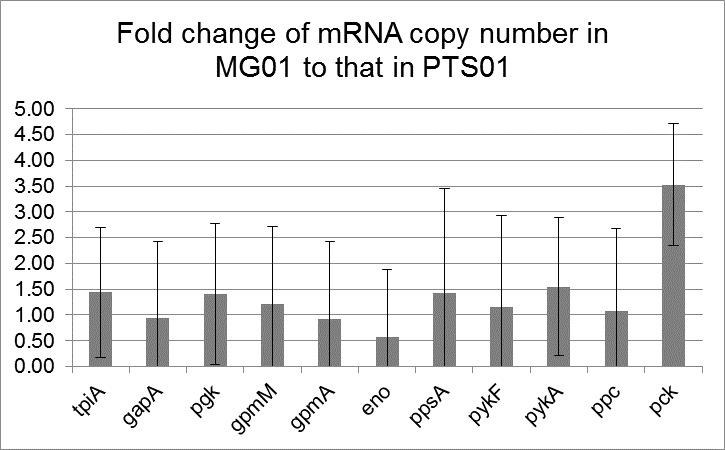


Figure S5. Fold change of transcriptional levels of genes in the pBAD-SIDF and pAC-crtEBI pathway of glycerol in PTS01 and MG01 grown in OPT1 media.

The growth media used in this study was OPT1 and all the samples of MG01 and PTS01 were collected at early log phase. The expression levels of all the genes were normalized to *cysG*


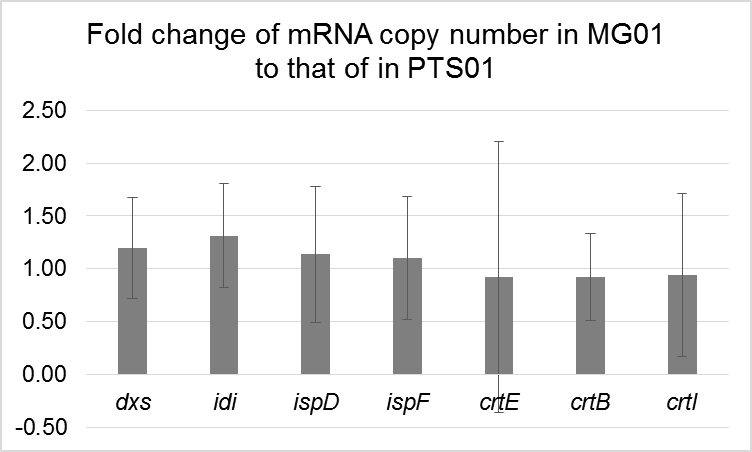

Supplement: File S2 — Figure S1, Fold change of transcriptional levels of dxs, ispE and crtE in PTS01 strain grown in OPT1 as compared to those in 2xPY medium and lycopene production. The transcriptional levels measured in 2xPY and OPT1 were compared at four different growth stages, late lag phase (0 h for 2xPY and 0 h for OPT1, the time of induction was set as time 0 h), early log phase (2 h for 2xPY and 4 h for OPT1), middle log phase (4 h for 2xPY and 10 h for OPT1) and late log phase (4 h for 2xPY and 10 h for OPT1). The sampling time in each growth stage was based on differences in the growth rates of PTS01, which was faster in 2xPY than in OPT1 (As shown in Figure 5.C). All the measurements were normalized to the expression of cysG. Figure S2, Fold change of plasmid copy number in PTS01 grown in OPT1 and 2xPY media. The x-axis described parameters identical to Figure S1 in File S2. The plasmid copy number was calculated using the copy number of a plasmid resistant gene (ampicillin resistant gene for pBAD-SIDF and chloramphenicol resistant gene for pAC-LYC) normalized by the copy number of the chromosomal gene ispE. Figure S3, Concentrations of metabolites and cofactors in PTS01 strain grown in 2xPY or OPT1 media. The metabolites in 2xPY and OPT1 were compared at three different growth stages, early log phase (3 h for 2xPY, 6 h for OPT1), middle log phase (6 h for 2xPY, 12 h for OPT1) and early stationary phase (12 h, 24 h). The time of induction was defined as time 0 h. Figure S4, Fold change of transcriptional levels of genes involved in the metabolic pathway of glycerol in PTS01 and MG01 grown in OPT1 media. The growth media used in this study was OPT1 and all the samples of MG01 and PTS01 were collected at the early log phase. The expression levels of all the genes were normalized to cysG. Figure S5, Fold change of transcriptional levels of genes in the pBAD-SIDF and pAC-crtEBI pathway of glycerol in PTS01 and MG01 grown in OPT1 media. The growth media used in this study was OPT [file pone.0075164.s002.doc]
